# Normal Values for the Two-Point Discrimination Test for Fingers in the Brazilian Population: Cross-Sectional Study

**DOI:** 10.1055/s-0046-1823633

**Published:** 2026-10-23

**Authors:** Mariana Ayumi Fujisaki, Vinícius Ynoe de Moraes, Fernando Araújo Pires, João Baptista Gomes dos Santos, Flávio Faloppa

**Affiliations:** 1 Hand Surgery Group, Department of Orthopedics and Traumatology, Escola Paulista de Medicina, Universidade Federal do Estado de São Paulo, São Paulo, SP, Brazil

**Keywords:** hand/surgery, neuropathy, semiology, mãos/cirurgia, neuropatia, semiologia

## Abstract

**Objective:**

To establish normative values for the two-point discrimination test in the Brazilian population.

**Methods:**

The present cross-sectional study was conducted in an adult population without neurological conditions affecting the hands. Discrimination was assessed on the fingertip pulp, from the thumb to the little finger, using a graded metal two-point discriminator with fixed tips. Normality was determined using the Von Frey test, with an esthesiometer consisting of calibrated nylon monofilaments.

**Results:**

A total of 984 hands, comprising 244 male and 248 female participants, were evaluated. The sample included 354 non-manual workers, 103 manual workers, and 35 retired participants. All fingers showed a discrimination range from 2 to 5 mm. The modes were 4 mm, 3 mm, 3 mm, 4 mm, and 4 mm for the 1 ^st^ , 2 ^nd^ , 3 ^rd^ , 4 ^th^ , and 5 ^th^ fingers, respectively.

**Conclusion:**

All fingers showed a discrimination interval between 2 and 5 mm. The mode was 4 mm for the 1st, 4th, and 5th fingers, and 3 mm for the 2nd and 3rd fingers.

## Resumo

**Objetivo:**

Determinar valores normativos para o teste de discriminação de dois pontos na população brasileira.

**Métodos:**

Estudo transversal em população adulta sem condições neurológicas que afetam as mãos. A discriminação (polpa digital, do polegar ao dedo mínimo) foi realizada com discriminador de dois pontos metálico, graduado e com pontas fixas. Para a determinação de normalidade, associou-se o teste de Von Frey (estesiômetro de monofilamentos de náilon calibrados).

**Resultados:**

Foram avaliadas 984 mãos, sendo 244 indivíduos do sexo masculino e 248 do sexo feminino. Quanto à ocupação, foram avaliados 354 trabalhadores não braçais, 103 braçais e 35 aposentados. Todos os dedos apresentaram intervalo de discriminação entre 2 e 5 mm. Dentre eles, a moda do 1 ^o^ , 4 ^o^ e 5 ^o^ dedos foi de 4 mm, a do 2 ^o^ e 3 ^o^ dedos foi de 3 mm.

**Conclusão:**

Todos os dedos apresentaram intervalo de discriminação mínima entre 2 e 5 mm e, dentre eles, a moda foi de 4 mm.

## Introduction

The two-point discrimination test defines the minimum distance required to distinguish between two distinct tactile stimuli. This test is widely used to assess the tactile acuity of a given cutaneous region and, consequently, its sensory function. 1 2 3

Despite being a readily accessible and low-cost test with broad clinical application, several aspects of the two-point discrimination test lack methodological standardization in the current literature. There are significant variations in application protocols, in the definition of distance intervals used, and in how results are obtained and interpreted, which compromise comparability across studies and between different healthcare settings. 4

Moberg 5 reported that he had discovered this technique 45 years earlier and that it was a major mistake not to have described it in detail at the time. The pressure required to perform the test is a key point of discussion, as it is extremely difficult to standardize the force applied by the examiner to generate sufficient tactile stimulus without causing pain and altering the test results. 5 6

There are two main approaches to determining the applied force. The first is the force sufficient for the patient to perceive the stimulus, which is subject to subjective interpretation and may lead to confusion with a painful stimulus. The second is the application of the minimum force necessary to cause a change in the color of the skin adjacent to the instrument's point of contact (a phenomenon known as blanching). 4 7

A computerized device for standardized pressure application was developed to overcome this difficulty. Although the device yielded good results, its application in clinical settings is limited. 8 Studies have also shown that changes in the intensity of the applied force may influence discrimination thresholds, indicating that stimulus pressure is an important determinant of the tactile acuity measured by the test. 7 9

However, differences in the perception of pressure applied with one or two points, rather than the tactile sensitivity to one or two points of contact, may influence the results even when the applied force is standardized across all measurements. 10 In addition, no consensus has been reached on the number of times each site should be assessed to obtain more reliable results, with recommendations ranging from 3 to 10 repetitions. 11 12

The classic reference value for the test considers < 6 mm as a normal result, 13 whereas more recent studies have expanded the spectrum, classifying results as normal (< 6 mm), fair (6–10 mm), and poor (11–15 mm). 14 Furthermore, gender and age influence normal values, 6 15 16 with performance declining in older subjects. 6 Testing frequency and repetition also affect discrimination results, facilitating the perception of two points at shorter distances. 17 Moreover, there are no studies evaluating whether regression occurs after prolonged periods without stimulation. Other potential influencing factors, such as hand dominance and occupation, have not been extensively evaluated. The present study aimed to establish the normative values of the two-point discrimination test in the Brazilian population.

## Methods

The present cross-sectional study was conducted at the Hand Surgery Group, Department of Orthopedics and Traumatology, Escola Paulista de Medicina, Universidade Federal do Estado de São Paulo, São Paulo, SP, Brazil. Data collection occurred between November 2023 and May 2024. The study was approved by the ethics committee under number CAAE: 69747623.4.0000.5505. This manuscript was prepared according to the Strengthening the Reporting of Observational Studies in Epidemiology (STROBE) guidelines, which are available at equatornetwork.com.

The population analysis included 492 healthy adult subjects (older than 20 years) without evident and/or reported conditions within the scope of hand surgery, most of whom were companions of patients attending the Hand Surgery Service at our hospital, totaling 984 hands.

The sample was divided into the following age groups: (1) 20 to 40 years, (2) 41 to 60 years, and (3) over 60 years. It was also categorized by gender (male or female) and type of occupational activity (manual vs. non-manual). The distribution included (1) 244 male subjects and (2) 248 female subjects. Regarding hand dominance and occupation, the sample consisted of 103 manual workers, 354 non-manual workers, and 35 retired subjects.

The Von Frey test was performed using an esthesiometer with calibrated nylon monofilaments for cutaneous sensitivity assessment to validate preserved neurological function ( Fig. 1 ). Only participants with green esthesiometry were eligible for the study.

**Fig. 1:**
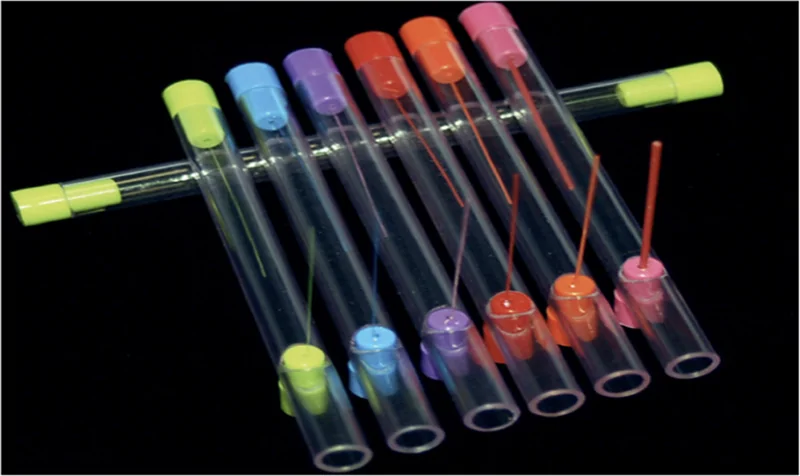
Esthesiometer used for the Von Frey test.

The test was performed with the participant seated, wrists in a neutral position, hands resting dorsally on the table, and fingers in a resting position. We used a graded metal two-point discriminator with fixed tips (suitable for assessing static tactile discrimination) ( Fig. 2 ). Measurements were taken on the fingertip pulp of each finger, from the 1st to the 5th, and the discriminator was positioned longitudinally along the midline of the fingertip pulp ( Fig. 3 ).

**Fig. 2:**
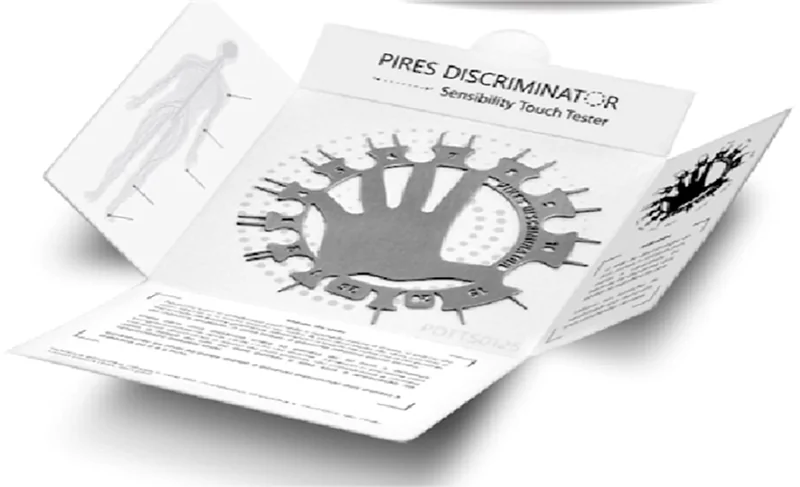
Metal two-point discriminator.

**Fig. 3:**
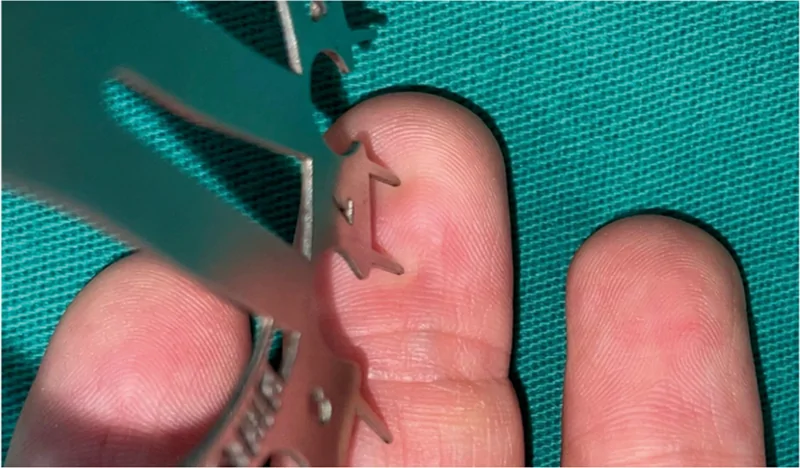
“Blanching” phenomenon described by Moberg.

The participants were asked to close their eyes to avoid visualizing the test. Contact was applied randomly, using either one or two points to ensure simultaneous contact with the skin. Participants were asked to indicate whether they perceived one or two points.

The initial measurement was set at 10 mm, decreasing progressively by 1 mm until the participant could no longer discriminate between two contact points. 13 The pressure applied to the fingertip pulp was sufficient to produce a change in skin color at the point of contact with the discriminator, a phenomenon described by Moberg as blanching ( Fig. 4 ). The recorded value corresponded to the last distance, in millimeters (mm), at which the participant could distinguish two points before reporting only one contact point.

**Fig. 4:**
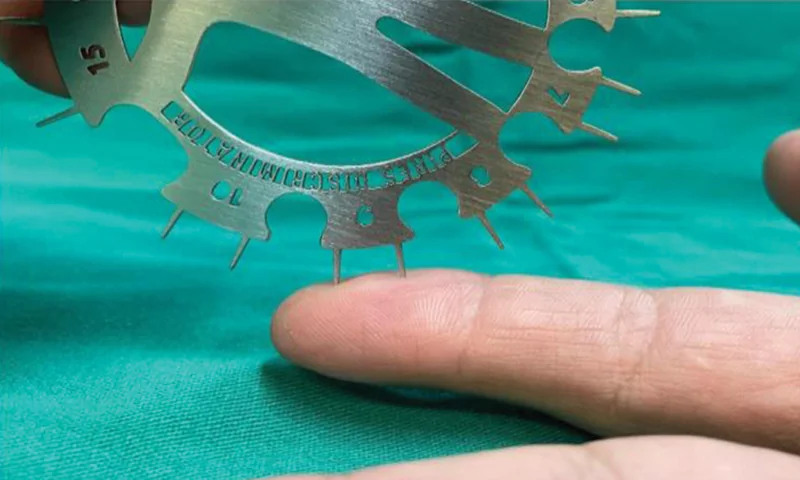
Measurement performed on the fingertip pulp of each finger, from the 1st to the 5th, with the discriminator positioned longitudinally along the midline of the fingertip pulp.

All data underwent statistical analyses to identify relevant correlations. Analysis of variance (ANOVA) was used to compare discrimination among fingers in the overall sample, with additional stratification by age group, sex, and occupation type. Paired Student's *t* -tests were used to compare the same fingers between the dominant and non-dominant hands. Tukey's multiple comparison method was used for pairwise finger comparison. The significance level was set at 5%.

## Results

The mode of the two-point distance in the Brazilian population was 4 mm. Figs. 5 6 show its distribution by finger.

**Fig. 5:**
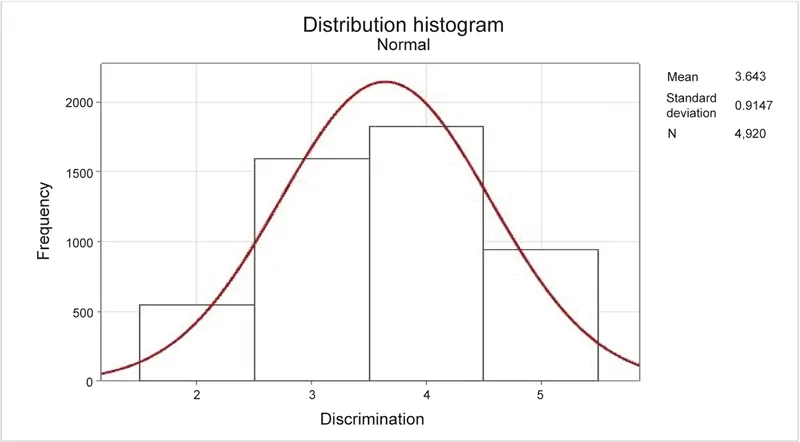
Distribution of the two-point distance by finger in the Brazilian population.

**Fig. 6:**
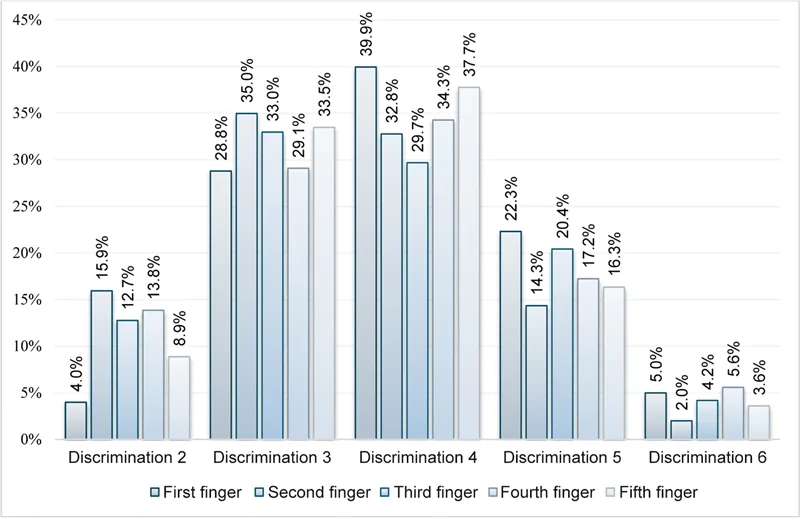
Distribution of two-point discrimination by finger (overall).

The means and their respective standard deviations, as well as the mode and confidence interval, are presented as follows: Table 1 for the Brazilian population; Table 2 according to age group; Table 3 for male and female genders; Table 4 according to the occupation of the evaluated subjects; and Table 5 according to hand dominance.

**Table 1:** Two-point discrimination by finger (n = 984)

		Minimum-maximum discrimination	Mean (CI95%)	Standard deviation	Mode	*p* -value
	1st finger	2–5	3,870 (3,819–3,921)	0.821	4	
	2nd finger	2–5	3,466 (3,408–3,524)	0.931	3	
All	3rd finger	2–5	3,612 (3,552–3,672)	0.960	3	< 0.001
	4th finger	2–5	3,624 (3,564–3,684)	0.957	4	
	5th finger	2–5	3,641 (3,588–3,694)	0.850	4	

**Table 2:** Two-point discrimination by age group

		Mean (CI95%)	Standard deviation	Mode	*p* -value
	1st finger	3,650 (3,589–3,711)	0.755	4	
	2nd finger	3,176 (3,110–3,242)	0.820	3	
20–40	3rd finger	3,282 (3,213–3,351)	0.855	3	< 0.001
	4th finger	3,370 (3,300–3,440)	0.865	3	
	5th finger	3,432 (3,370–3,494)	0.771	3	
	1st finger	4,097 (3,991–4,203)	0.853	4	
	2nd finger	3,794 (3,679–3,909)	0.928	4	
41–60	3rd finger	3,992 (3,877–4,107)	0.926	5	< 0.001
	4th finger	3,883 (3,758–4,008)	1.005	4	
	5th finger	3,863 (3,749–3,977)	0.916	4	
	1st finger	4,382 (4,269–4,495)	0.690	5	
	2nd finger	4,097 (3,955–4,239)	0.872	5	
≥ 61	3rd finger	4,313 (4,183–4,443)	0.797	5	< 0.001
	4th finger	4,222 (4,083–4,361)	0.848	5	
	5th finger	4,118 (3,995–4,241)	0.753	4	

**Table 3:** Two-point discrimination by gender

		Mean (CI95%)	Standard deviation	Mode	*p* -value
	1st finger	3,911 (3,836–3,986)	0.855	4	
	2nd finger	3,522 (3,441–3,603)	0.921	3	
Female	3rd finger	3,677 (3,596–3,758)	0.917	4	< 0.001
	4th finger	3,679 (3,596–3,762)	0.943	4	
	5th finger	3,641 (3,564–3,718)	0.876	4	
	1st finger	3,828 (3,758–3,898)	0.784	4	
	2nd finger	3,410 (3,327–3,493)	0.940	3	
Male	3rd finger	3,545 (3,456–3,634)	0.998	3	< 0.001
	4th finger	3,568 (3,482–3,654)	0.970	4	
	5th finger	3,641 (3,568–3,714)	0.823	4	

**Table 4:** Two-point discrimination by occupation

		Mean (CI95%)	Standard deviation	Mode	*p* -value
	1st finger	4,248 (4,154–4,342)	0.686	4	
	2nd finger	3,990 (3,868–4,112)	0.894	4	
Manual worker	3rd finger	4,121 (4,002–4,240)	0.872	5	< 0.001
	4th finger	4,117 (4,005–4,229)	0.818	4	
	5th finger	4,015 (3,900–4,130)	0.841	4	
	1st finger	3,701 (3,642–3,760)	0.802	4	
	2nd finger	3,237 (3,175–3,299)	0.843	3	
Non-manual worker	3rd finger	3,363 (3,298–3,428)	0.887	3	< 0.001
	4th finger	3,400 (3,333–3,467)	0.915	3	

**Table 5:** Two-point discrimination by dominance

		Mean (CI95%)	Standard deviation	Mode	*p* -value
	Dominant	3,868 (3,791–3,945)	0.873	4	
1 ^st^ finger					0.914
	Non-dominant	3,872 (3,804–3,940)	0.767	4	
	Dominant	3,437 (3,353–3,521)	0.954	3	
2 ^nd^ finger					0.124
	Non-dominant	3,496 (3,416–3,576)	0.908	3	
	Dominant	3,591 (3,508–3,674)	0.943	3	
3 ^rd^ finger					0.304
	Non-dominant	3,632 (3,546–3,718)	0.977	4	
	Dominant	3,606 (3,520–3,692)	0.976	4	
4 ^th^ finger					0.410
	Non-dominant	3,642 (3,559–3,725)	0.939	4	
	Dominant	3,602 (3,527–3,677)	0.849	4	
5 ^th^ finger					0.021
	Non-dominant	3,681 (3,606–3,756)	0.850	4	

All fingers showed a discrimination range from 2 to 5 mm. The mode was 4 mm for the 1 ^st^ , 4 ^th^ , and 5 ^th^ fingers and 3 mm for the 2 ^nd^ and 3 ^rd^ fingers.

Among all fingers evaluated, the second finger showed shorter discrimination distances compared to the others.

Age-related variation was observed ( Table 2 ). The youngest group (20–40 years), totaling 296 subjects, showed shorter discrimination distances compared to the 124 subjects aged 41 to 60 years and the 72 subjects older than 60.

The 354 non-manual workers showed better discrimination capacity than the 103 manual workers, whereas the retired subjects, totaling 35, showed the lowest capacity ( Table 4 ). There was no significant difference in discrimination capacity between the 244 men and 248 women, and between dominant and non-dominant hands, occurred only in the fifth digit ( Tables 3 5 ).

## Discussion

Considering the lack of standardization in the Brazilian population, the current study evaluated 984 non-neuropathic hands, demonstrating values from 2 to 5 mm. These values corroborate previous findings, 18 with a mode of 4 mm for the 1 ^st^ , 4 ^th^ , and 5th fingers and 3 mm for the 2 ^nd^ and 3 ^rd^ fingers. The Von Frey monofilament test was used to ensure preserved neurological function, and the study included only subjects with a green esthesiometry. 19 20

Despite the clinical exclusion criteria, the monofilament test has limitations, including variable sensitivity for early neuropathies and potential failures in detecting subclinical abnormalities. 21 Furthermore, variability in its diagnostic accuracy has been described, with the possibility of false negatives and false positives when used as a standalone screening method. 22 Therefore, the inclusion of mild, undetected sensory abnormalities cannot be completely ruled out.

The second finger showed shorter discrimination distances, indicating greater tactile sensitivity, potentially related to the higher density of receptors in distal regions and those with more frequent functional use. 23 The more richly innervated the finger, the more clearly and accurately it can perceive the difference between two contact points. 18 23

The correlation between increasing age and poorer sensory performance corroborates previous findings in the literature, which describe a progressive decline in sensitivity with aging. 16 The ability to discriminate two points tends to deteriorate over time, a phenomenon associated with the quantitative and functional reduction of cutaneous receptors, especially Meissner corpuscles and Merkel cells. 16 Classical histological studies by Cauna and Ross 24 and Ridley 25 demonstrated this age-related decrease, providing morphological and functional support for the observation of higher discrimination thresholds in older subjects.

There was no significant difference between genders. This finding is partially consistent with the literature, as a study conducted in the United States found that women had lower scores than men ( *p* < 0.001). 26 These authors observed higher thresholds in women, as well as better performance at the fingertips. 26 The lack of statistical significance regarding hand dominance corroborates the findings of Cheng. 14 Thus, it is suggested that dominance does not have a significant impact on two-point discrimination.

In addition, methodological variations among studies, such as applied force, instrument orientation, response criteria, and examiner training, contribute to discrepancies in the results reported in the literature. The methodological standardization adopted in the current study, including the use of the Von Frey monofilament test to exclude subclinical neuropathies, allowed the definition of more consistent and specific values for the Brazilian population, ranging from 2 to 5 mm. These findings refine and corroborate the classical literature.

The main limitation of the present study is its cross-sectional design, which prevents causal or longitudinal analyses, as well as the scarcity of recent studies on test standardization in healthy populations. Nevertheless, the classical physiological and methodological foundations remain valid. Future studies, with more diverse samples and standardized methodologies, may expand and update the proposed normative parameters.

## Conclusion

Two-point discrimination in the Brazilian population with healthy hands from 2 to 5 mm, with a mode of 4 mm. The mode was 4 mm for the 1 ^st^ , 4 ^th^ , and 5 ^th^ fingers, and 3 mm for the 2nd and 3 ^rd^ fingers. Younger participants, aged 20 to 40 years, demonstrated shorter discrimination distances compared to older age groups. Non-manual workers showed better two-point discrimination capacity compared to manual workers, followed by retired subjects.

## References

[1] Saulicz E Saulicz O Koterba J Sikora D Saulicz A Saulicz M Inter- and Intra-Examiner Reliability Study of Two-Point Discrimination Test (TPD) and Two-Point Estimation Task (TPE) in the Sacral Area of Pain-Free Individuals Diagnostics (Basel) 2023 13 22 3438 10.3390/diagnostics13223438 PMC10670713 37998574

[2] Tong J Mao O Goldreich D Two-point orientation discrimination versus the traditional two-point test for tactile spatial acuity assessment Front Hum Neurosci 2013 7 579 10.3389/fnhum.2013.00579 PMC3772339 24062677

[3] Taylor S C Atkinson G Dixon J Robinson J R Ryan C G Test-retest reliability of the two-point discrimination test on the sole of the foot in people with multiple sclerosis Physiol Meas 2020 41 11 11NT01 10.1088/1361-6579/abc4c6 33105127

[4] Adamczyk W Luedtke K Szikszay T Two-point discrimination test: the time has come for standardization. International Association for the Study of Pain (IASP), Jan. 25,2026. Acesso em: Feb. 11, 2026 from:https://www.iasp-pain.org/publications/relief-news/article/two-point-discrimination-standardization/

[5] Moberg E Two-point discrimination test. A valuable part of hand surgical rehabilitation, e.g. in tetraplegia Scand J Rehabil Med 1990 22 03 127 134 2244189

[6] John A A Rossettie S Rafael J Cox C T Ducic I Mackay B J Clinical Assessment of Pain and Sensory Function in Peripheral Nerve Injury and Recovery: A Systematic Review of Literature Arch Plast Surg 2022 49 03 427 439 10.1055/s-0042-1748658 PMC9142258 35832158

[7] Franco P G Bohrer R CD Rodacki A LF Reprodutibilidade intra-avaliador do teste de discriminação de dois pontos na sola dos pés em idosos e jovens assintomáticos Braz J Phys Ther 2012 16 06 523 527 10.1590/S1413-35552012005000062

[8] Tustumi F Nakamoto H A Tuma P Junior Milcheski D A Ferreira M C Prospective study on tactile sensitivity in the hands of a Brazilian population using the pressure-specified sensory device Rev Bras Ortop 2015 47 03 344 347 10.1016/S2255-4971(15)30109-9 PMC4799424 27042644

[9] Jung J K Byun J S Choi J K The Effect of Applied Force on Two-Point Discrimination Threshold in the Trigeminal Region J Oral Facial Pain Headache 2019 33 04 371 376 10.11607/ofph.2362 31247059

[10] Bell-Krotoski J A Buford W L Jr The force/time relationship of clinically used sensory testing instruments J Hand Ther 1997 10 04 297 309 10.1016/S0894-1130(97)80045-2 9399179

[11] Omer G E Bell-Krotoski J Sensibility testing London Saunders 1998 11 28

[12] Dellon A L Sensibility: Re-education of sensation in the hand Baltimore Williams & Wilkins 1981

[13] Gelberman R H Urbaniak J R Bright D S Levin L S Digital sensibility following replantation J Hand Surg Am 1978 3 04 313 319 10.1016/s0363-5023(78)80030-6 681714

[14] Cheng A Evaluation of functional sensibility in the local Chinese population Hong Kong J Occup Ther 1999 10 01 3 11

[15] Koo J P Kim S H An H J Two-point discrimination of the upper extremities of healthy Koreans in their 20's J Phys Ther Sci 2016 28 03 870 874 10.1589/jpts.28.870 PMC4842456 27134375

[16] Shimokata H Kuzuya F Two-point discrimination test of the skin as an index of sensory aging Gerontology 1995 41 05 267 272 10.1159/000213693 8537010

[17] Faloppa F Leite N M Propedêutica ortopédica e traumática São Paulo Artmed 2013

[18] Bell-Krotoski J A Light touch-deep pressure testing using the Semmes-Weinstein monofilaments St. Louis CV Mosby 1989 42

[19] Von Frey M RF Verspätete Schmerzempfindungen Z Gesamte Neurol Psychiatr 1922 79 324 333. Available from:https://link.springer.com/article/10.1007/BF02878457

[20] Dros J Wewerinke A Bindels P J Van Weert H C Accuracy of monofilament testing to diagnose peripheral neuropathy: a systematic review Ann Fam Med 2009 7 06 555 558 10.1370/afm.1016 PMC2775618 19901316

[21] Dunker Ø Uglem M Bu Kvaløy M Diagnostic accuracy of the 5.07 monofilament test for diabetes polyneuropathy: influence of age, sex, neuropathic pain and neuropathy severity BMJ Open Diabetes Res Care 2023 11 06 e003545 10.1136/bmjdrc-2023-003545 PMC10660161 37989346

[22] Weber V EH Uber den Tastsinn. Arch Anat Physiol Wiss Med 1835 152 160. Available from:https://www.biodiversitylibrary.org/item/49216#page/7/mode/1up

[23] Lundy-Ekman L Neuroscience: Fundamentals for Rehabilitation. 3rd ed St. Louis Elsevier 2007

[24] Cauna N Ross L L The fine structure of Meissner's touch corpuscles of human fingers J Biophys Biochem Cytol 1960 8 02 467 482 10.1083/jcb.8.2.467 PMC2224947 13691669

[25] Ridley A Silver staining of nerve endings in human digital glabrous skin J Anat 1969 104(Pt 1):41 48 PMC1232029 4178184

[26] Maheswari K U Devi R G Priya A J Estimation of Tactile Sensation by Two Point Discrimination Among 18 Years Old People J Pharm Res Int 2021 33(47B):355 363 10.9734/jpri/2021/v33i47B33135

